# Supplementary material for: Blockchain enabled policy-based access control mechanism to restrict unauthorized access to electronic health records
Source: PeerJ Comput Sci. 2025 Jan 23;11:e2647. doi: 10.7717/peerj-cs.2647 (PMC11784709; doi:10.7717/peerj-cs.2647)
Supplement: Supplemental Information 2 [file peerj-cs-11-2647-s002.pdf]

Algorithm 1: Policy for storing and managing patient data

```
P_i = {{Policy for storing and managing patient data, v1.0},
(1, {patient, controller}, {whole},
{store_data, check_logs, revoke_consent}, 1),
{proof_document, verification_method}}
Input: SubjectID, LoginRequest, CheckPolicy
Output: Action
Action = "Signup Required"
if ValidUser(SubjectID) AND CheckPolicyDetails then
    Action = "Login Successful"
    function subject_checkup()
    if ControllerDetails() and FollowSubjectPolicy() then
        StartCheckupOfPatient()
        MedicalRecommendation()
        StoreData()
        SuggestTest()
        PatientConsent()
    end if
    end function
    function Change_Consent()
    if PatientWantsToChangeConsentType() then
        SelectConsentType()
        Print "Select the Types."
    end if
    end function
    function Revoke_Consent()
    if PatientWantsToRevokeConsent() then
        DataUsage = "None"
    end if
    end function
    function Check_Logs()
    if UserDetailsAreValid() then
        Display(HistoryOfMedicalDetails)
        Print "History of Medical Details"
    end if
    end function
else
    function Signup()
    Print "signup"
    Register(SubjectDetails)
    Print "Subject Details"
    end function
end if
```

Algorithm 2: Controller Policy for Data Management

$P_c = \{\{\text{Managing patient data storage and AC}, \text{v2.0}\},$
$(1, \{\text{controller, patient}\}, \{\text{consent\_type, compliance}\},$
$\{\text{verify, store, access\_logs, process\_revoc,}$
$\text{receive\_access\_requests, manage\_audits}\}, 1),$
$\{\text{proof\_document}, \text{verification\_method}\}\}$

Input: SubjectID, ControllerID, RequesterID, Data
Output: Action
Action = ”Signup Required”
if ValidUser(ControllerID) **then**
    Action = ”Login Successful”
    ManageDataStorage()
    EnsurePatientConsent()
    ProvideAccessToConsentLogs()
    ProcessConsentRevocation()
    HandleAccessRequests()
    ManageAuditsAndLogs()
else
    Signup()
end if
ManageDataStorage
if ValidUser(SubjectID) **then**
    StartCheckupOfPatient()
    MedicalRecommendation()
    SuggestTest()
    StoreData()
    PatientConsent()
end if
Providing_consent_logs
if ValidUser(SubjectID) **then**
    Action = ”Logs Provided”
end if
ProcessConsentRevocation
if ValidUser(SubjectID) **and** FollowSubjectPolicy() **then**
    Action = ”Revoke Consent”
end if
HandleAccessRequests
if ValidUser(RequesterID) **and** FollowSubjectPolicy() **then**
    Action = ”Access Granted”
end if
ManageAuditsAndLogs
if ValidUser(SubjectID) **and** FollowSubjectPolicy() **then**
    Action = ”Access Granted”
end if
ConductAudits
Action = ”Audits and logs managed by Controller”

Algorithm 3: Requester Access to Patient Data

$P_{\text{req}} = \{\{\text{Policy for requesting access to patient data, v3.0}\},$
$(1, \{\text{req, con}\}, \{\text{purpose\_justify, compliance}\},$
$\{\text{submit\_request, justify\_purpose, adhere\_audit\_log}\}, 1),$
$\{\text{proof\_document, verification\_method}\}\}$
Input: RequesterID, ControllerID, Purpose
Output: Action
if ValidUser(ControllerID) **then**
    Action = ”Login Successful”
    ProvideAccessToData(RequesterID, RequestDetails, FollowSubjectPolicy)
    LogAuditResults(RequesterID)
else
    Signup()
end if
if ProvideAccessToData(RequesterID, RequestDetails, FollowSubjectPolicy) **then**
    Action = ”Access Granted”
    ProvideAccessToData(RequesterID, RequestDetails)
    LogAuditResults(RequesterID)
else
    Action = ”Invalid User”
end if
